# Flaw-induced plastic-flow dynamics in bulk metallic glasses under tension

**DOI:** 10.1038/srep36130

**Published:** 2016-10-25

**Authors:** S. H. Chen, T. M. Yue, C. P. Tsui, K. C. Chan

**Affiliations:** 1 Advanced Manufacturing Technology Research Centre, Department of Industrial and Systems Engineering, The Hong Kong Polytechnic University, Hung Hom, Kowloon, Hong Kong

## Abstract

Inheriting amorphous atomic structures without crystalline lattices, bulk metallic glasses (BMGs) are known to have superior mechanical properties, such as high strength approaching the ideal value, but are susceptible to catastrophic failures. Understanding the plastic-flow dynamics of BMGs is important for achieving stable plastic flow in order to avoid catastrophic failures, especially under tension, where almost all BMGs demonstrate limited plastic flow with catastrophic failure. Previous findings have shown that the plastic flow of BMGs displays critical dynamics under compression tests, however, the plastic-flow dynamics under tension are still unknown. Here we report that power-law critical dynamics can also be achieved in the plastic flow of tensile BMGs by introducing flaws. Differing from the plastic flow under compression, the flaw-induced plastic flow under tension shows an upward trend in the amplitudes of the load drops with time, resulting in a stable plastic-flow stage with a power-law distribution of the load drop. We found that the flaw-induced plastic flow resulted from the stress gradients around the notch roots, and the stable plastic-flow stage increased with the increase of the stress concentration factor ahead of the notch root. The findings are potentially useful for predicting and avoiding the catastrophic failures in tensile BMGs by tailoring the complex stress fields in practical structural-applications.

The plastic deformation of bulk metallic glasses (BMGs), a new class of amorphous alloys with superior mechanical properties compared with conventional crystalline alloys, is accommodated by intermittent bursts of shear avalanches^1^,^2^, which also widely occur in other solids in releasing energy, such as in earthquakes^3^, collapse of sandpiles^4^, and dislocation gliding in crystals^5^,^6^,^7^. Without the confinement of periodic crystalline lattices, BMGs demonstrate catastrophic failures by the rapid propagation of shear bands (shear avalanches), hindering the practical structural-applications of BMGs^8^. In some BMG specimens with relatively larger plasticity, where the catastrophic failures are delayed, the plastic flow exhibit a power-law scaling and tuned criticality under compression^9^,^10^,^11^,^12^. Understanding the plastic-flow dynamics of BMGs is significant in achieving stable plastic flow to avoid catastrophic failures. For example, the use of multiple samples^13^ and complex stress fields^14^ can drive the chaotic plastic flow to evolve to a critical dynamics to delay such catastrophic failures. The critical plastic-flow dynamics of BMGs can also be obtained by tailoring sample geometries (aspect ratio)^15^, testing temperatures^16^ and applied strain rates^16^. However, differing from the dislocation-mediated plastic deformation of crystals, where the critical dynamics were observed under both compression^7^,^17^ and tension tests^5^,^18^, the plastic-flow dynamics of BMGs under tension have not been reported. In view of the fact that some classes of BMGs have certain or even large plasticity under compression, but almost all classes of BMGs have very limited ductility leading to catastrophic failures under tension, understanding of the plastic-flow dynamics of BMGs under tension is extremely vital before widespread applications of BMGs as structural materials can be achieved.

Although BMGs have high strength approaching the theoretical value^19^, some of them exhibit high fracture toughness^20^,^21^ when compared with conventional brittle materials, such as ceramics. For instance, Demetriou *et al*.^20^ have reported a Pd_79_Ag_3.5_P_6_Si_9.5_Ge_2_ (at.%) BMG with an unprecedented combination of toughness and strength in amorphous materials, where the toughness and strength are usually mutually exclusive in natural materials^21^. With better capability to shield the propagation of cracks than conventional brittle materials, BMGs demonstrate good flaw tolerance under tension^22^,^23^. The sharp notches can even strengthen and toughen BMGs simultaneously by stress-driven structural re-ordering^24^. The findings of the deformation behavior of flawed BMGs under tension^22^,^23^,^24^,^25^,^26^,^27^,^28^,^29^,^30^,^31^ suggest that it might be possible to achieve increased plastic flow in flawed BMGs under tension, and to give an insight into the corresponding plastic-flow dynamics. Here, by the use of tailored single-side-notched BMGs, we show that the critical dynamics of power-law scaling in plastic flow can also be achieved in tensile BMGs. Moreover, differing from the plastic flow of BMGs under compression, the flaw-induced plastic flow under tension show an upward trend in the amplitudes of load drops with time, resulting in the formation of a stable plastic-flow stage with a power-law distribution of the load drop.

## Results

### Tensile testing results

**Figure 1:**
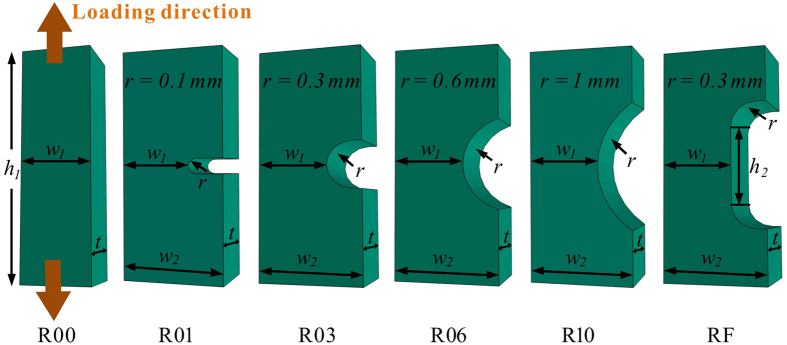
Reduced sections of the flawed tensile BMG specimens. The schematic diagram showing the reduced sections of the notched tensile specimens, where *h*_1_ = 10 mm, *w*_1_ = 0.8 mm, *w*_2_ = 1.2 mm, *t* = 0.8 mm, and *h*_2_ = 1 mm.

**Figure 2:**
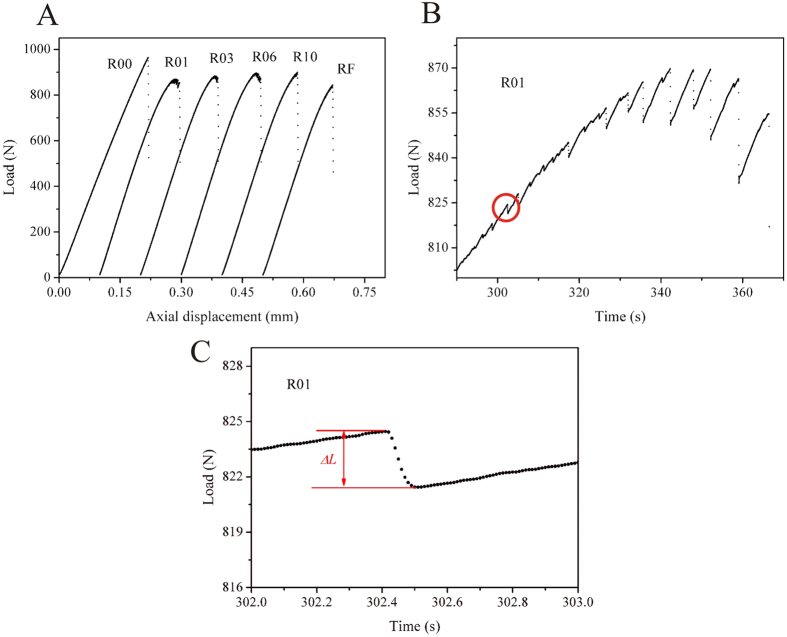
Tensile testing results. (**A**) The load-axial displacement curves. (**B**) Serrated flow in the R01 specimen. (**C**) An example of a load drop in the R01 specimen, as indicated by the circle in (**B**).

**Figure 3:**
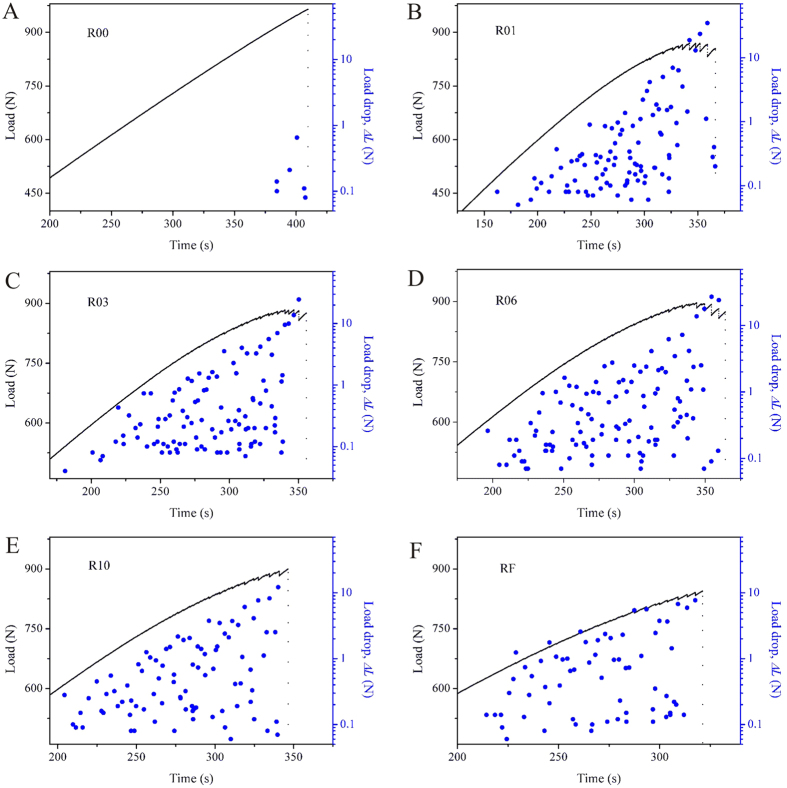
Load drops with the increase of time. (**A**–**F**) Flow serrations and corresponding load drops of the single-side-notched BMG specimens.

The schematic diagram in Fig. 1 shows the reduced sections of the single-side-notched Zr_57_Cu_20_Al_10_Ni_8_Ti_5_ (at.%) BMG specimen with tailored notches. The conventional tensile specimen and five kinds of notched specimens are denoted as R00, R01, R03, R06, R10 and RF, respectively. The specimens R01, R03, R06 and R10 have curved notch bottoms with varying radii, and the RF specimen has a flat notch bottom with a width of *h*_2_. The tensile testing results are given in Fig. 2. The conventional tensile specimen (R00) fractured at a nominal strain of 2.24 ± 0.02%, which is in line with previous findings^32^. In contrast, serrated flow of the load drops (*ΔL*) was observed in the notched specimens by examining the load-axial displacement curves at a higher magnification, as shown in the example in Fig. 2(B,C). The collected load drops of the specimens are further plotted against time, in Fig. 3. In the conventional tensile specimen (R00), several load drops were observed before catastrophic failure occurred. Small load drops before the catastrophic failure were also observed in previous findings on BMGs^20^. However, such slip events with an average of 0.1% plastic strain do not change the brittle nature of BMGs leading to catastrophic failure. For the single-side-notched BMG specimens, a large number of load drops were observed, as can be seen in Fig. 3(B–F). The displacement range for plastic flow (with the presence of load drops) was determined as *d*_p_ = *d*_f_−*d*_e_, where *d*_f_ and *d*_e_ are the axial displacement at fracture and the point with the first presence of a load drop, respectively. The *d*_p_ values of the notched specimens are 0.095 ± 0.014, 0.093 ± 0.001, 0.088 ± 0.002, 0.077 ± 0.001, and 0.063 ± 0.011 mm for R01, R03, R06, R10 and RF specimens, respectively. The observation of a certain range of displacement for plastic flow and a large number of load drops in the notched specimens provide sufficient plastic flow data for examination of the plastic-flow dynamics.

The authors would like to point out that the normalized ultimate tensile strength (NUTS) of the notched BMGs is smaller than the conventional tensile specimens (R00), which is different from the MD simulations of the Cu_50_Zr_50_ metallic glasses^23^. Such a discrepancy may result from the sample size effect with different deformation mechanisms, where the deformation mechanism transits from localized deformation with catastrophic failures to relatively homogeneous deformation with plasticity when sample size is reduced^33^,^34^. At the macroscopic scale in the present work, the stress relaxation at the notch roots cannot relieve the stress concentration as shown in the MD simulations to cause the insensitive effect^23^. Similar phenomenon has also been observed in the Fe-P metallic glasses^29^.

### Power-law scaling of the plastic flow

**Figure 4:**
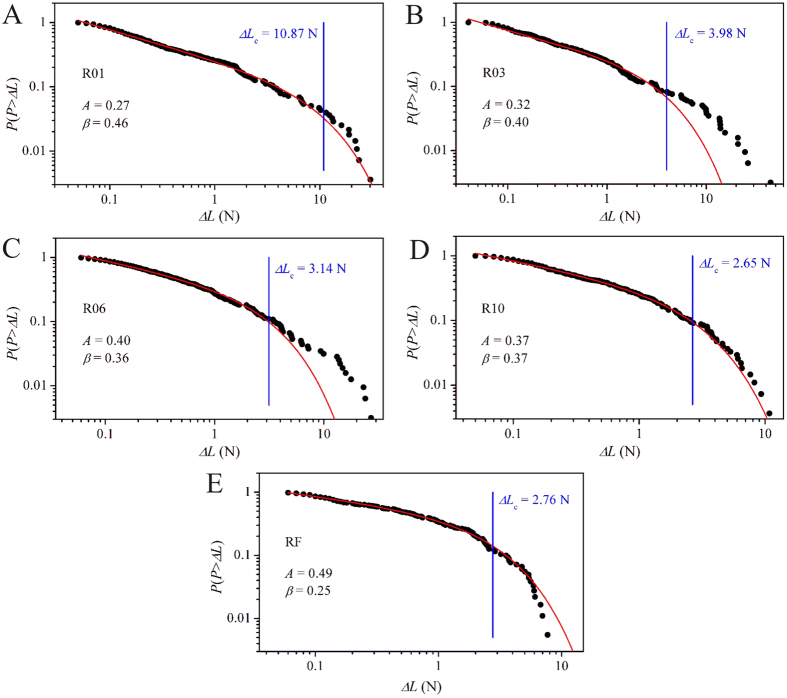
Statistical results. (**A**–**E**) The cumulative probability distributions of the load drops of the single-side-notched BMG specimens. The red lines are the power-law fitted curves, where the load drops (*ΔL*) follow a power-law distribution till the cut-off values *ΔL*_c_ (vertical blue lines). The fitting parameter, *Adj. R-Square*, for specimens R01 to RF are 0.996, 0.992, 0.996, 0.996, and 0.998, respectively.

With the collected load drop data, the plastic-flow dynamics of the single-side-notched BMG specimens were investigated using statistical analysis. The cumulative probability distributions of the load drops, *P*(*P > ΔL*), were fitted using a power-law scaling function, *P* (*P > ΔL*) = *A* (*ΔL*)^−*β*^ exp(−*ΔL*/*ΔL*_c_), where *A* is a normalized constant and *β* a scaling exponent. The cut-off load drop, *ΔL*_c_, denotes the threshold value at which the decay of the power-law scaling would occur. The fitted results of the specimens are shown in Fig. 4. It can be seen that the plastic flow of the single-side-notched BMG specimens can also be well modeled using a power-law decay function, as shown in the compression tests^9^,^10^,^11^,^12^. This suggests that the power-law scaling of the plastic-flow dynamics can also be used to describe the plastic flow in tensile BMG specimens, similar to crystalline alloys^5^,^18^. The profile of the shear avalanches in the single-side-notched tensile specimens is dependent on the size and shape of the notches. With increased notch radius, the specimens have an overall increasing trend in the constant *A* and a decreasing trend in the constant *β*, and the RF specimen with flat notch root has the largest *A* value and the smallest *β* (Fig. 4). It has been reported that Zr-based BMGs have particular values of the constants *A* (≈9.5) and *β* (≈0.40) under compression^9^. The change of the constants *A* and *β* in the single-side-notched specimens indicates that the shear avalanche profile can be affected by the change of the notch size and shape, which usually occurs in the specimens with different composition^9^,^35^, or under an external disturbance^9^. Additionally, the cut-off load drop (*ΔL*_c_) decreases as the notch radius increases.

As shown in Fig. 3, an upward trend of the increase of the amplitudes of the load drops was observed in the single-side-notched specimens. The upward trend of the amplitudes of the load drops with small increasing rate has been widely observed in BMG compression tests, and such trend is known to have no significant effect on the plastic-flow dynamics and can be ignored during statistical analysis of the plastic-flow dynamics^13^,^15^,^36^. However, in the present single-side-notched specimens, the upward trend has a large increasing rate, which follows a power-law scaling. As can be seen in Fig. S1 in Supplementary Materials, the load drops of the notched specimens are well fitted using a power-law equation *ΔL* = exp(*λ* + δ*t), where *λ* and *δ* are fitting parameters. More importantly, this trend is independent of the notch size and shape. This phenomenon has not been reported in the plastic flow of BMGs under compression tests. With the upward trend of the amplitudes of the load drops, the cut-off load drop (*ΔL*_c_) can then be regarded as an indicator for the formation of a stable stage of plastic flow. When *ΔL < ΔL*_c_, the distribution of the load drops follows a power-law scaling, *D* ~ *ΔL*^−*μ*^, where D is the numbers of load drops and *μ* is a fitting parameter (see Supplementary Fig. S2). Only when the amplitude of the load drop exceeds the value of *ΔL*_c_, the plastic flow transits from the power-law scaling to decay, and results in the unpredictable plastic flow associated with catastrophic failure. If we note the displacement at which a load drop larger than the cut-off value *ΔL*_c_ occurs for the first time as *d*_s_, the plastic flow with axial displacement (*d*_a_) less than such value, i.e., *d*_a_ < *d*_s_, can be regarded as a stable plastic-flow stage. We therefore define a parameter, *d*_*p*-s_, = *d*_s_−*d*_e_, as the axial displacement for stable plastic flow, where *d*_e_ is the axial displacement for the first presence of a load drop (in following sections, *d*_*p*-s_ is noted as the displacement for stable plastic flow). The *d*_*p*-s_ values of the notched specimens are 0.084 ± 0.012, 0.066 ± 0.003, 0.059 ± 0.004, 0.051 ± 0.002, 0.043 ± 0.010 mm, for the R01, R03, R06, R10 and RF specimens respectively. It was determined that all the single-side-notched BMG specimens have stable plastic-flow stages, and the magnitudes vary with different notch sizes and shapes.

### Effect of stress gradients on the plastic flow

**Figure 5:**
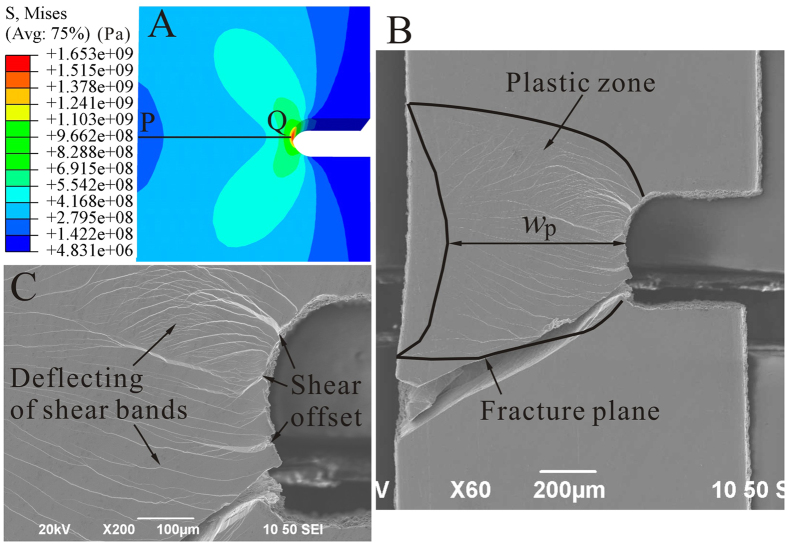
Stress distribution and the plastic zone of the R01 specimen. (**A**) FEM results of the stress distribution around the notches, where P-Q indicates the symmetric plane. (**B**) SEM image of a fractured specimen. (**C**) The shear band distribution ahead of the notch root at a higher magnification.

The plastic deformation of BMGs is accommodated by the initiation and propagation of shear bands, which are significantly dependent on the applied stress fields^2^,^37^. In the single-side-notched BMG specimens, the stress distributions around the notches changed from uniform tensile stresses to complex stress states under the applied loading, which were simulated using FEM analysis, as shown in Fig. 5 and Supplementary Figs S3–S6. (In this work, the stress gradient describes the gradient stress distribution from the stress concentration region around the notch root to the unyield region far away from the notch^38^.) It can be seen that the R01, R03 and R06 specimens have fishtail-shaped stress concentration regions around the notch roots, while in the R10 and RF specimens, the stress concentration regions change to fan-shaped regions. The corresponding plastic zones of the fractured specimens, where shear bands distributed, are also given in Fig. 5 and Supplementary Figs S3–S6, respectively, and are in line with the FEM predictions. BMGs are reported to have a good combination of high strength and high toughness, and are able to shield the propagation of cracks. The flaw-induced plastic flow in the present findings are related to the shielding of the propagation of the cracks. The plastic zone size of the present BMGs can be estimated using the equation *r*_p_ = *K*_c_^2^/π*σ*_y_^2 ^20. With *σ*_y_ = 1701 MPa (the average value for the R00 specimens), and the fracture toughness of *K*_c_ = 69 MPa m^1/2^ (The value of a BMG with a similar composition of Zr_52.5_Cu_17.9_Al_10_Ni_14.6_Ti_5_^39^), the plastic zone radius *r*_p_ was estimated at 0.52 mm. If we take the plastic zone width (*w*_p_ in Fig. 5 and Supplementary Figs S3–S6) ahead of the notch root as *r*_p_^40^, the experimental observations (0.55–0.65 mm) are in line with the predictions. With the effective width of the notched specimens *w*_1_ = 0.8 mm (Fig. 1), the plastic zone size *r*_p_ suggests that such BMG is intrinsically capable of shielding the propagation of cracks in the present BMG specimens. Nevertheless, the change of the plastic zone shape suggests that the shielding-ability is also affected by the extrinsically introduced stress gradients.

**Figure 6:**
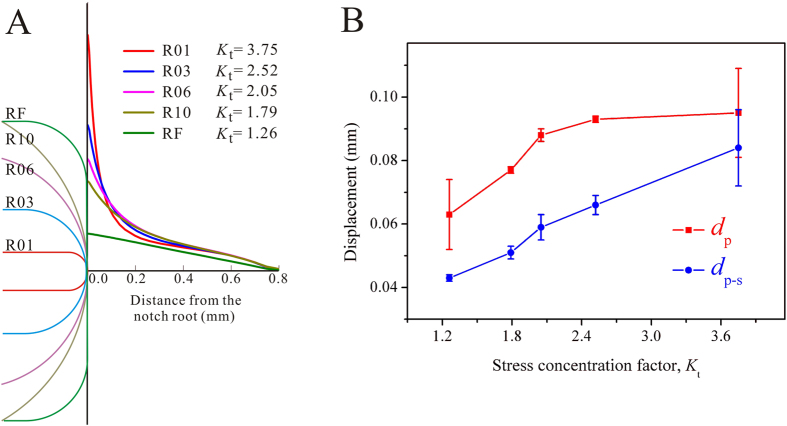
Stress concentrations. (**A**) Stress concentration ahead of the notch roots (along the symmetric planes). (**B**) Relationships between the stress concentration factors and the displacement for plastic flow (*d*_p_) and stable plastic flow (*d*_p-s_).

To characterize the effect of the stress gradients, the stress concentrations ahead of the notch roots are shown in Fig. 6A. It can be seen that the stress decreases significantly away from the notch roots, and the rate decreases with an increase of the notch radii, with the RF specimen having the smallest rate at decreasing. The stress concentration factor of the notched specimens, *K*_t_ = *σ*_max_/*σ*_nominal_, was also calculated and is shown in Fig. 6A, where *σ*_max_ is the peak stress at the notch root and *σ*_nominal_ is the nominal stress of the net section^41^. With increasing notch radii in the R01 to R10 specimens, the stress concentration factors (*K*_t_) decrease from 3.75 to 1.79, and the RF specimen with a flat notch root has the smallest value of 1.26. With the increase of the stress concentration factor *K*_t_, the displacement for plastic flow (*d*_p_) and stable plastic flow (*d*_p-s_) also increase (Fig. 6B), indicating the occurrence of more plastic flow and a wider range of the stable plastic-flow stages.

The decrease of stress concentration factors results in the gradual change of the stress concentration regions from fishtail-shaped to fan-shaped, as shown in Fig. 5 and Supplementary Figs S3–S6. In the stress concentrated regions, curved shear bands were observed, which initiated from the notch root and propagated along the stress concentrated-regions, and were then deflected back to the symmetric plane of the specimens (see an example in Fig. 5C). A large number of curved shear bands were observed in the R01 and R03 specimens (Fig. 5C and Supplementary Fig. S3C), fewer in the R06 and R10 specimens (Supplementary Figs S4C and S5C), and several curved shear bands were observed in the RF specimens (Supplementary Fig. S6B). As compared with the R00 specimen without notches, the presence of the curved shear bands is the result of shielding the crack propagation, where more deflection of the shear bands indicates a higher shielding capability^20^,^26^,^42^. On the other hand, the curved shear bands are also able to deflect the fracture path of the specimens to dissipate more energy to form more plastic flow. It can then be speculated that a larger stress concentration factor could result in more plastic flow in the tensile BMG specimens. When the stress concentration factor (*K*_t_) decreases, fewer curved shear bands will be formed and even the fracture path can transit from a deflected plane to a straight one, similar to the R00 specimen (Supplementary Fig. S7). Typically, the R01 specimen has not only a large number of curved shear bands but also obvious shear offsets at the notch roots (Fig. 5C), which are associated with the occurrence of more plastic flow. While for the RF specimen with the least plastic flow (smallest *d*_p_), it only has a few curved shear bands initiating from the stress concentration regions (Supplementary Fig. S6B). Ahead of the notch root, it has several straight shear bands (Supplementary Fig. S6C), and, not surprisingly, it fractured along a straight path with an angle of about 53°, which is almost the same as the R00 specimen (Supplementary Fig. S7). With the change of stress gradients from R01 to RF specimens, i.e., the decreasing of the stress concentration factor (*K*_t_), the capability to shield the propagation of shear bands also decreases. A higher order of stress gradient (a larger *K*_t_) can lead to a stronger barrier for the propagation of shear bands, and delay the catastrophic failures by blunting the crack tips^20^ and deflecting the fracture planes^43^, resulting in the occurrence of larger load drops. Therefore, it is reasonable to obtain a higher cut-off load drop in the fitting results of the specimens with higher orders of stress gradients. Regarding the upward trend in the load drops of the plastic flow, a higher cut-off value implies the occurrence of more plastic flow before the catastrophic failures (Fig. 6B).

With the presence of stress gradients (stress concentrations), the yield regions of the notched specimens evolve during the loading process, resulting in the upward trend of the amplitudes of the load drops in the plastic flow. Taking the R01 specimen for example, the evolution of the yield regions is shown in Supplementary Fig. S8. Under applied loading, the yield regions serve as the origins for the shear bands and the unyield regions impede the propagation of shear bands^2^,^38^. At the beginning, yielding only occurs at a small region ahead of the notch root (Supplementary Fig. S8A), where the propagation of the shear bands will be immediately stopped by the unyield regions^38^. The elastic energy released during such a process is very limited, resulting in relatively smaller load drops^9^. When the loading process proceeds (Supplementary Fig. S8B,C), the enlargement of the yield regions can delay the impediment effect from the unyield regions to release more elastic energy, resulting in larger load drops in the load-axial displacement curves^9^. The upward trend of load drops is also in line with the evolution of shear bands. At the stable plastic-flow stages, the smaller load drops are related to the formation of multiple shear bands, where relatively smaller shear offsets are formed. When the deformation evolves to a stage beyond the stable plastic flow, the increased load drops result in the formation of larger shear offsets (Fig. 5C). At this stage, the propagation of shear bands cannot be impeded by stress gradients and cause the formation of cracks, leading to catastrophic failures.

## Discussion

Stemming from the liquid-like atomic structures, BMGs have mechanical properties superior to conventional crystalline alloys. However, such unique atomic structures also make most BMGs more susceptible to catastrophic failure than conventional crystalline alloys, especially under tension^19^,^44^. The present findings show that the plastic flow of BMGs can also display a power-law scaling under tension by tailoring flaws. The critical dynamics of the plastic flow suggest that the catastrophic failure of BMGs under tension could be avoided or delayed by driving the serrated plastic flow to evolve to a critical state. On the other hand, although many previous studies have been devoted to enhance the plastic deformation behavior of BMGs under tension by tailoring flaws^24^,^30^,^31^,^45^,^46^, due to the variations of the mechanical properties^47^,^48^,^49^,^50^,^51^, how to determine the stable plastic-flow stages and to predict the catastrophic failures in flawed specimens is still challenging and unknown. The flaw-induced plastic flow in this work shows an upward trend of the amplitudes of the load drops, which follow a power-law scaling. This enables the appearance of stable plastic-flow stages with load drops less than the cut-off value (*ΔL*_c_), where the distribution of the load drops follows a power-law scaling, and the catastrophic failures only occur beyond the stable stages with load drops larger than *ΔL*_c_. Such characteristic of the plastic flow is beneficial for predicting and avoiding catastrophic failures in BMGs^13^,^14^, and this has not been previously reported for the compression of BMGs. In addition, in practical applications of BMGs as structural materials, they always deform under complex stress states^52^,^53^,^54^,^55^,^56^. The present findings of the flaw-induced plastic flow in BMGs not only give a deeper insight into the deformation mechanisms of BMGs under complex fields, i.e., gradient stress distributions around the notches, but also provide guidance in tailoring the plastic deformation in BMGs through geometry design, i.e., by tuning the sizes and shapes of the notches. Although BMGs are considered as poised for wide structural applications^8^, the practical applications of BMGs and BMG structures under tensile loadings are still limited, which are mainly due to the catastrophic failures under tension. The present findings of stable plastic flow, achieved by designing complex stress fields in tensile specimens, are potentially useful for designing BMG structures and achieving a desirable tensile performance in BMGs/BMG structures for practical structural-applications.

Since the burst of avalanches in BMGs is path dependent and it is also challenging to capture detailed information of the initiation and propagation of shear bands accurately using FEM analysis^57^,^58^, the present results have limitations that the quantitative relationships between the amplitudes of the load drops, the evolution of the yield regions, and activation of the shear bands have not been established, and are worthy of further investigation. Additionally, Chen *et al*.^40^ have reported that the fracture toughness of metallic glasses changes as the decrease of the notch radius till a critical value, and becomes notch radius-independent when the notch radius is less than the critical value. It implies that, when the notch radius in the present work is further decreased at relatively larger *K*_t_ values, i.e., larger than 3.75, the *d*_p_ and *d*_p-s_ values may also become notch radius-independent. The evolution of the *d*_p_ values in Fig. 6B has already shown a decreasing trend in increasing rate when *K*_t_ reaches to 3.75. This is also a limitation of the present work and further investigations may be devoted to examine the relationships between the plastic flow and the stress concentration factor when the *K*_t_ values are larger than the present results.

## Methods

### Sample preparation

Master alloy ingots of a nominal composition of Zr_57_Cu_20_Al_10_Ni_8_Ti_5_ (at.%) were fabricated by melting the pure elements of Zr (99.8%), Cu (99.999%), Al (99.99%), Ni (99.999%) and Ti (99.995%) under a Ti-gettered Ar atmosphere. After remelting the alloy ingots five times, as-cast BMG specimens with 3 mm diameter were produced by suction casting the alloy ingots into water-cooled copper moulds. The amorphous atomic structures of the BMG specimens were confirmed using standard X-ray diffraction (XRD) analysis. The single-side-notched tensile specimens, as shown in Fig. 1, were fabricated using electrical discharge machining (EDM) on an FI 240 SLP wire-cut EDM machine. The side surfaces of the notched specimens were polished into a mirror finish using abrasive paper with grits up to 2,000.

### Mechanical test

Tensile tests of the notched specimens were conducted on an Instron 5565 Material Testing Machine at a loading rate of 0.06 mm/min. Three specimens were tested for each condition. To analyze the serrated plastic flow, 100 data points per second were recorded. The number of load drops collected for examining the critical dynamics were 278 (R01), 316 (R03), 317 (R06), 274 (R10) and 182 (RF), respectively. After the mechanical testing, the side surfaces were examined on a Jeol JSM-6490 scanning electron microscope.

### FEM analysis

FEM analysis was employed to characterize the gradient stress distributions around the notches in an elastic state. A commercial ABAQUS package was used with input material parameters as: 1.701 GPa for the yield strength (taken from the R00 specimen), 79.5 GPa for Young’s modulus (also taken from the R00 specimen), and 0.36 for Poisson’s ratio^59^. The stress concentration factors (*K*_t_) of the specimens were calculated using FEM under a static state. The peak stresses (*σ*_max_) were collected directly from the FEM results, and the nominal stresses (*σ*_nominal_) were obtained by integrating the stresses along the cross sections. Although many studies attempted to simulate the plastic deformation of BMGs, however, a constitutive model which can accurately capture the fine details of shear banding in BMGs has yet to be reported^57^,^58^. In this work, we use an ideal elastic-plastic constitutive model to simulate the evolution of the yield regions of the notched specimens during tensile testing. Although such model cannot capture the formation and propagation of shear bands, it is good to characterize evolution of yield regions where shear bands are formed^32^,^60^.

## Additional Information

**How to cite this article**: Chen, S. H. *et al*. Flaw-induced plastic-flow dynamics in bulk metallic glasses under tension. *Sci. Rep*. **6**, 36130; doi: 10.1038/srep36130 (2016).

## Supplementary Material

Supplementary Information
