# Supplementary material for: Flaw-induced plastic-flow dynamics in bulk metallic glasses under tension
Source: Sci Rep. 2016 Oct 25;6:36130. doi: 10.1038/srep36130 (PMC5078772; doi:10.1038/srep36130)
Supplement: Supplementary Information [file srep36130-s1.pdf]

Supplementary materials for

Flaw-induced plastic-flow dynamics in bulk metallic glasses under tension

S.H. Chen[1]*, T.M. Yue[1], C.P. Tsui[1] and K.C. Chan[1]

[1]Advanced Manufacturing Technology Research Centre, Department of Industrial and Systems Engineering, The Hong Kong Polytechnic University, Hung Hom, Kowloon, Hong Kong

* Corresponding author. E-mail: s-h.chen@polyu.edu.hk, cshunhua@gmail.com

This supplementary information file includes:

Fig. S1. Power-law fitting of the load drops against time.

Fig. S2. Power-law fitting of the load drops at the stable plastic-flow stages.

Fig. S3. Stress distribution and the plastic zone of the R03 specimen.

Fig. S4. Stress distribution and the plastic zone of the R06 specimen.

Fig. S5. Stress distribution and the plastic zone of the R10 specimen.

Fig. S6. Stress distribution and the plastic zone of the RF specimen.

Fig. S7. SEM image of a fractured R00 specimen.

Fig. S8. The evolution of yield regions in the R03 specimen.

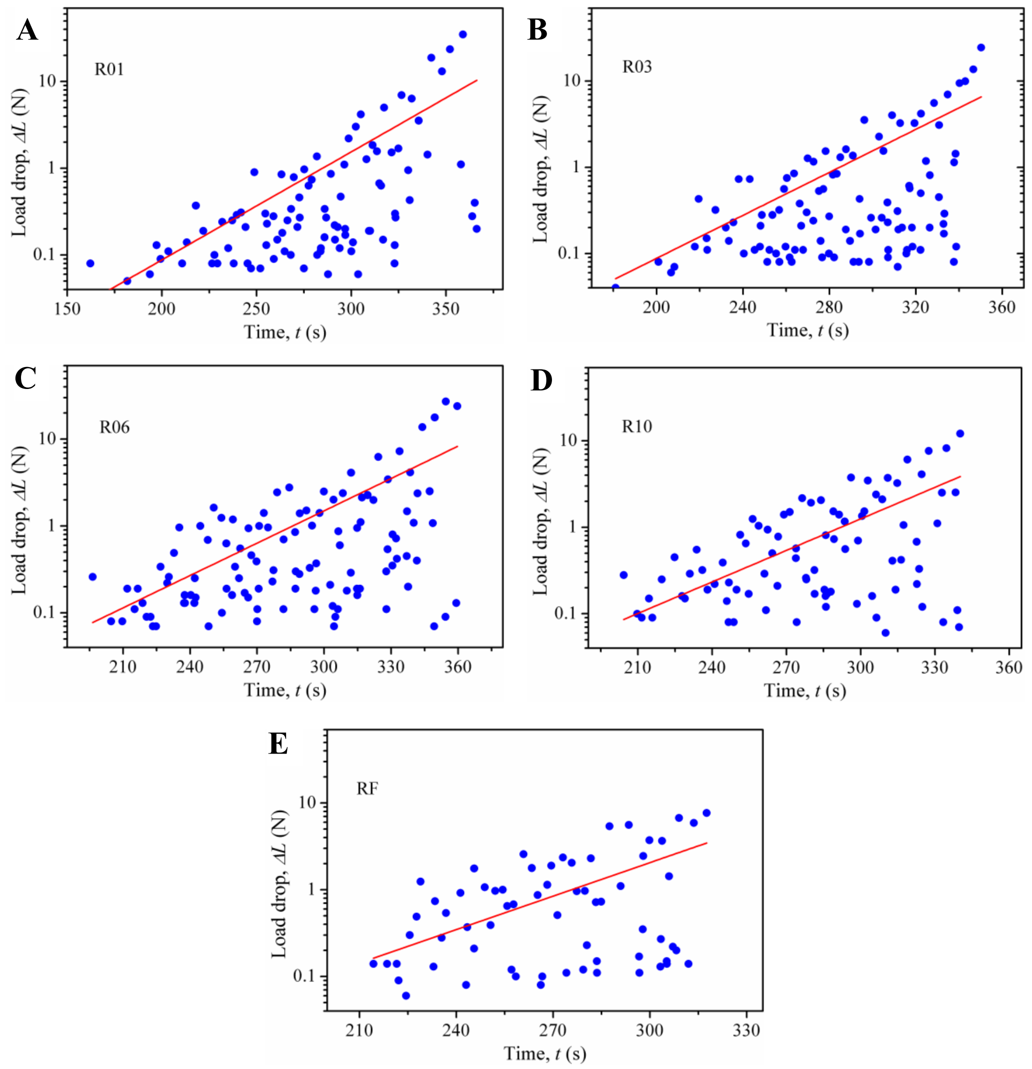

Fig. S1. Power-law fitting of the load drops against time. (**A-E**) The increase of load drops (blue dots) against time of the notched specimens was fitted using a power-law equation (red line) $\Delta L = \exp(\lambda + \delta^* t)$, where $\lambda = -8.17$ and $\delta \approx 0.029$.

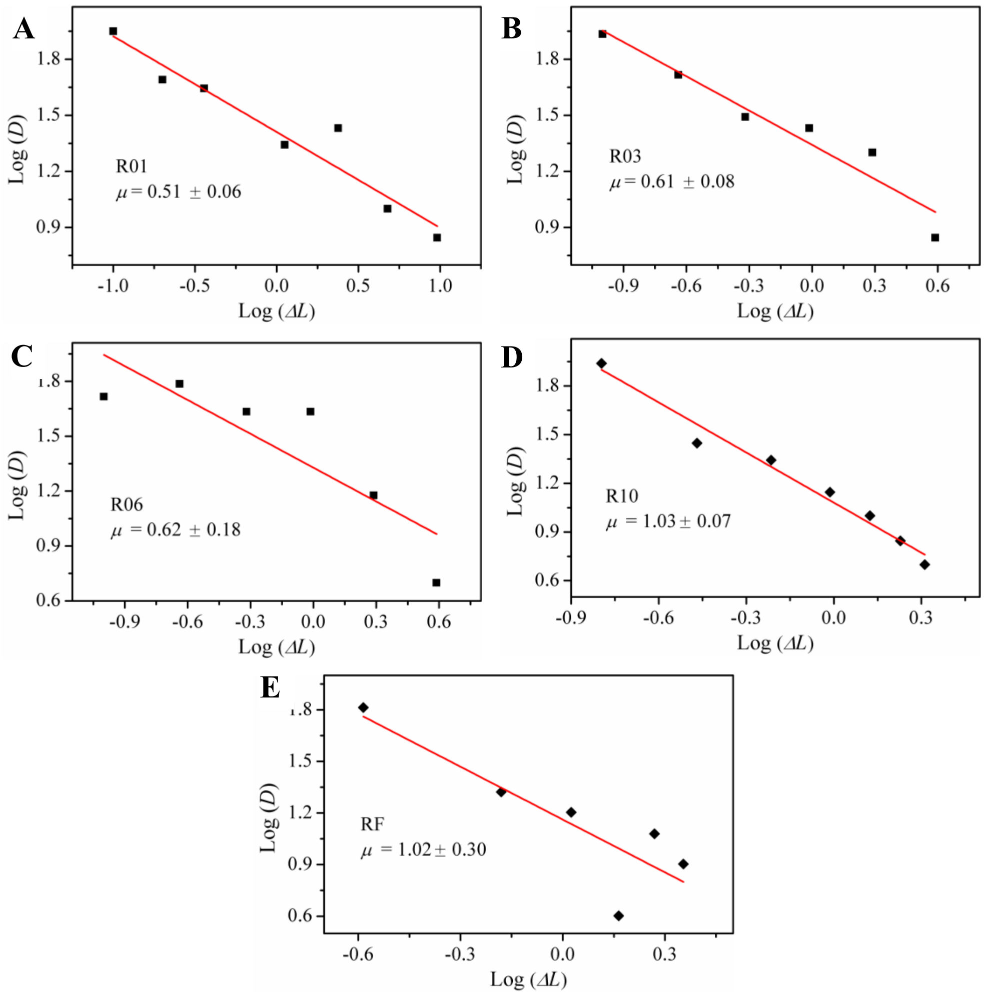

Fig. S2. Power-law fitting of the load drops at the stable plastic-flow stages. (A-E) The distributions of the numbers (D) of load drops (ΔL) at the stable plastic-flow stages, i.e., within $d_{p\text{-}s}$, were fitted using a power-law equation $D \sim \Delta L^{-\mu}$. It can be seen that at the stable plastic-flow stages, the distributions of the load drops of the single-side-notched BMG specimens follow a power-law scaling.

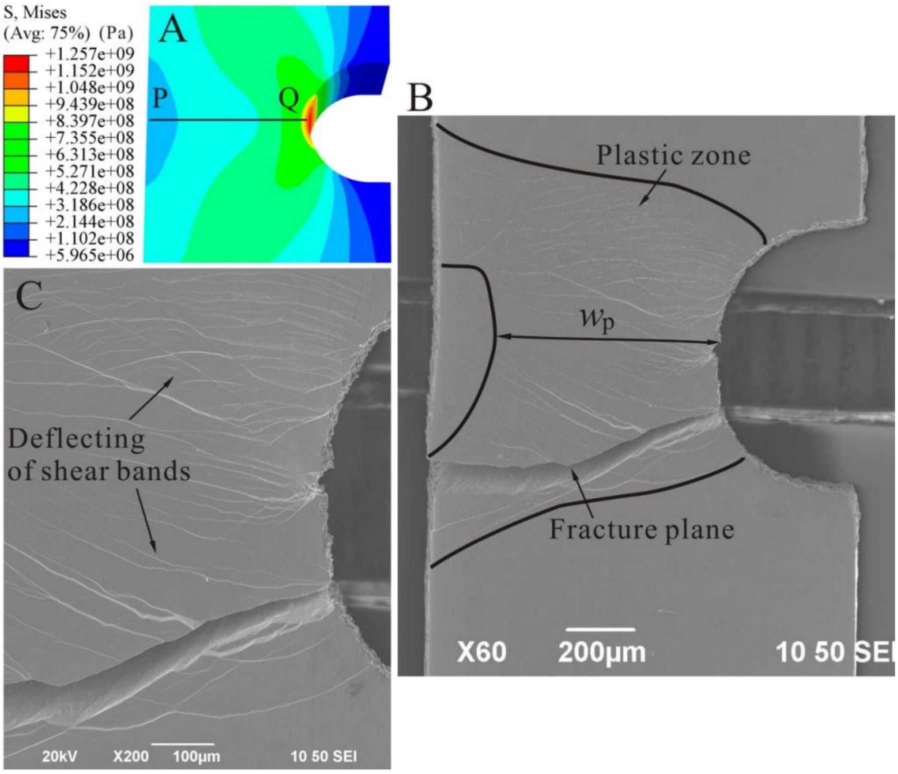

Fig. S3. Stress distribution and the plastic zone of the R03 specimen. (**A**) FEM results of the stress distribution around the notches, where P-Q indicates the symmetric plane. (**B**) SEM image of a fractured specimen. (**C**) The shear band distribution ahead of the notch root at a higher magnification.

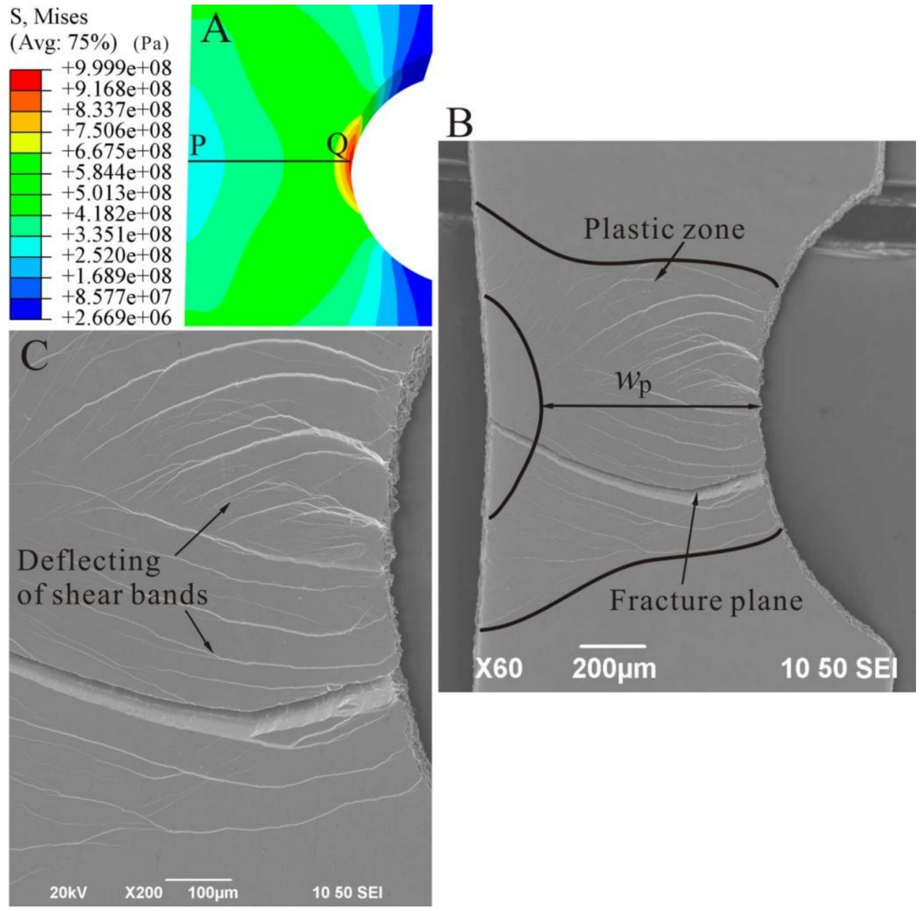

Fig. S4. Stress distribution and the plastic zone of the R06 specimen. (**A**) FEM results of the stress distribution around the notches, where P-Q indicates the symmetric plane. (**B**) SEM image of a fractured specimen. (**C**) The shear band distribution ahead of the notch root at a higher magnification.

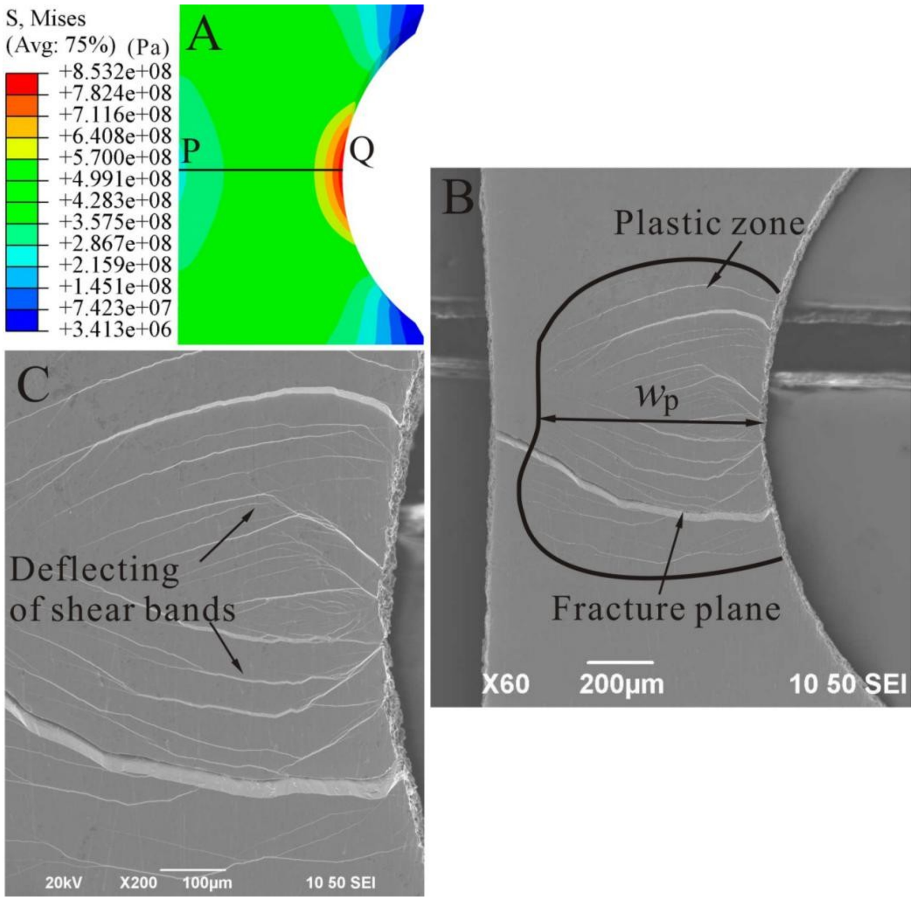

Fig. S5. Stress distribution and the plastic zone of the R10 specimen. (**A**) FEM results of the stress distribution around the notches, where P-Q indicates the symmetric plane. (**B**) SEM image of a fractured specimen. (**C**) The shear band distribution ahead of the notch root at a higher magnification.

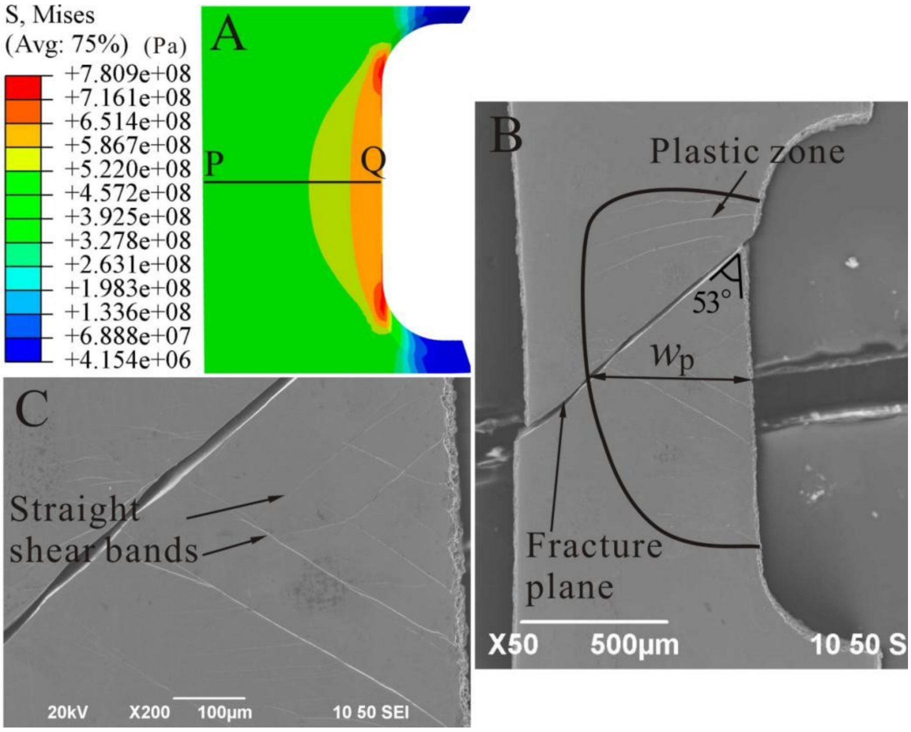

Fig. S6. Stress distribution and the plastic zone of the RF specimen. (**A**) FEM results of the stress distribution around the notches, where P-Q indicates the symmetric plane. (**B**) SEM image of a fractured specimen, where the specimen fractured at an angle of about 54°. (**C**) The shear band distribution ahead of the notch root at a higher magnification, where several straight shear bands were observed.

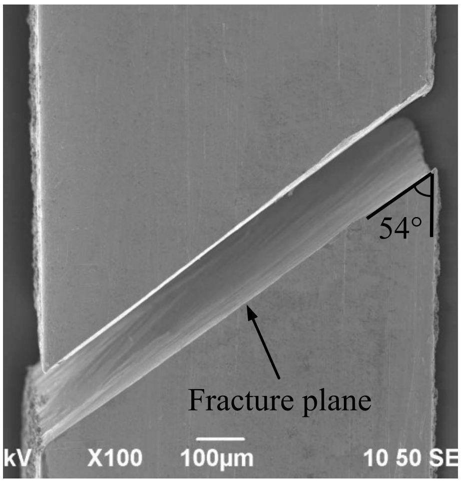

Fig. S7. SEM image of a fractured R00 specimen. This specimen fractured at an angle of about 54°.

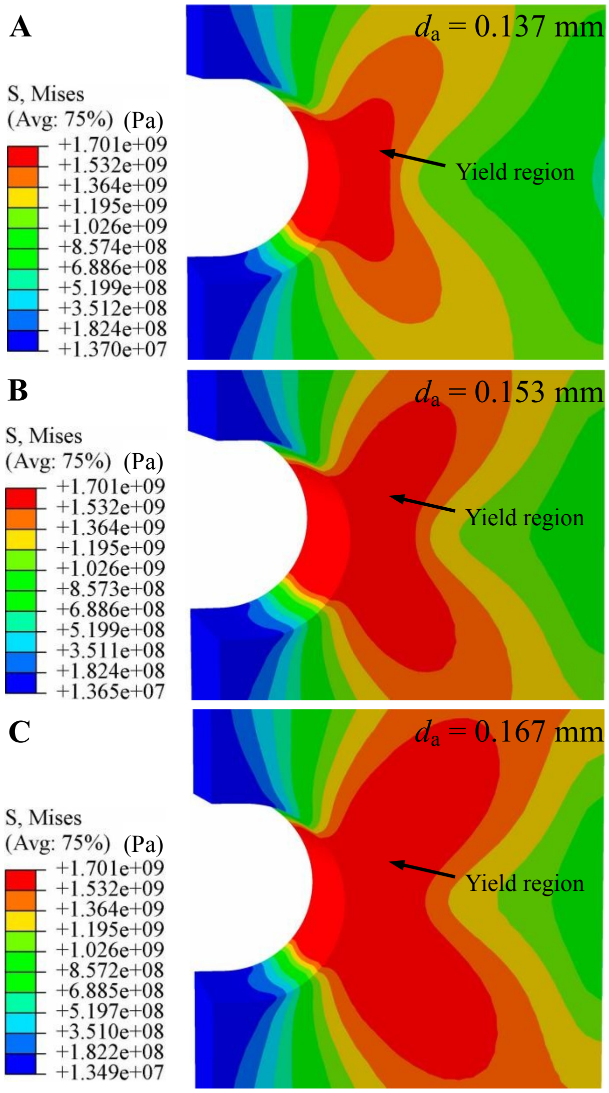

Fig. S8. The evolution of yield regions in the R03 specimen. (**A-C**) FEM results showing the evolution of the yield regions in the R03 specimen at varying axial displacements (d_a).
